# Interventions for children and adolescents with specific learning disability and co-occurring disorders

**DOI:** 10.1038/s41390-025-04261-0

**Published:** 2025-07-18

**Authors:** Daniel R. Espinas, Sharon Vaughn, Lynn S. Fuchs

**Affiliations:** 1 The University of Texas at Austin, Austin, TX USA; 2 Vanderbilt University, Nashville, TN USA; 3 American Institutes for Research, Arlington, VA USA

## Abstract

**Abstract:**

Interventions for individuals with specific learning disabilities (SLD) largely focus on performance within a single performance domain (e.g., reading). However, it is more the rule than the exception that individuals with SLD will experience difficulties that span multiple domains (e.g., reading and mathematics), and that co-occur with other disorders (e.g., attention-deficit/hyperactivity disorder). In this article, we discuss what has been learned about supporting children and adolescents with SLD affecting multiple performance domains (i.e., reading and mathematics), and with common co-occurring difficulties with attention and anxiety. We also identify opportunities for future research for students with disabilities in math and reading and/or other disorders, e.g., attention-deficit/hyperactivity disorder.

**Impact:**

- It is common for children and adolescents with specific learning disabilities in reading to present with co-occurring difficulties with mathematics, attention, and anxiety.
- Compared to children and adolescents whose difficulties are isolated to reading, those with co-occurring difficulties with mathematics, attention, and anxiety often present with more pronounced difficulties in each affected domain.
- Combined interventions (e.g., reading instruction and anxiety management) show promise for addressing co-occurring learning difficulties.
- Further research is needed to examine the efficacy and feasibility of interventions for children and adolescents with co-occurring difficulties.

## Introduction

Specific Learning Disability (Given our focus on school-based interventions, we use the *term specific learning disability*, as defined in the Individuals with Disabilities Education Improvement Act of 2004. Throughout the article, we will treat this term as synonymous with *learning disability*, *learning disorder*, *specific learning disorder*, and *developmental learning disorder*, as defined in other diagnostic systems and research studies.) (SLD) is among the most common mental health conditions affecting children and adolescents,^1^ with prevalence estimated at ~3.6–9.7% in the U.S. school-aged population.^2,3^ It has long been understood that SLD can affect performance in multiple academic domains (e.g., reading, mathematics)^4–6^ and frequently co-occurs with other neurodevelopmental disorders (e.g., attention-deficit/hyperactivity disorder [ADHD])^1,7–12^ and health conditions (e.g., anxiety).^1,13^ Indeed, individuals with an SLD in reading often present with a co-occurring SLD in mathematics.^5,14–19^ Moreover, those with an SLD in math are more than twice as likely as those with no SLD to present with a co-occurring SLD in reading.^6^ Estimates of co-occurrence range widely though (e.g., 17%^19^ to 64%^18^) depending on the measures and identification criteria that are used.^15,20^ Similarly, ~40% of children and adolescents with an SLD also present with ADHD.^21^ Moreover, compared to individuals without an SLD, those with an SLD present with more anxiety symptoms (*d* = 0.61)^22^ and are more likely to develop anxiety disorders.^12^

Despite these common patterns of co-occurrence, SLD intervention research has largely focused on addressing performance within single academic domains, often without documenting whether students have comorbidities or investigating how these may impact outcomes.^5,23,24^ Considerably less attention has been paid to developing and evaluating interventions for those with more complex presentations.^23^ In this review, we discuss what has been learned about how to effectively support individuals with SLDs in reading and three common co-occurring difficulties: mathematics, attention, and anxiety.

## Causes and outcomes

Subtypes of SLD (e.g., dyslexia, dyscalculia) are associated with distinct behavioral phenotypes, each with difficulties restricted to performance in particular domains (e.g., reading, mathematics) and subdomains (e.g., word-level reading, math problem-solving). Features of these phenotypes are associated with unique (domain-specific) and shared (domain-general) causal factors distributed and connected within and between the levels of cognition, neural systems, and genetics.^25,26^ Performance within a domain is influenced through transactions between the individual and their environment.^27^ Through this flow, biological vulnerabilities and environmental risks can increase one’s likelihood of developing an SLD.^28–30^ Conversely, biological endowments and protective environments can counteract risks, promoting growth and reducing the pattern and severity of an individual’s difficulties.^29,31,32^ The causes of SLD may be understood then to be dynamic, multifactorial, and probabilistic.^26,33^

Various explanations may account for why two (or more) disorders co-occur.^34–38^ Disorders may co-occur merely by chance or artifactually represent different manifestations of the same underlying condition.^35^ Alternatively, disorders may co-occur because of correlated liabilities^39^ and shared risk factors.^26^ For instance, the phenotypic characteristics of dyslexia (i.e., word-level reading and spelling difficulties)^40,41^ and ADHD (i.e., attention difficulties and/or hyperactivity-impulsivity)^41^ are associated with a panoply of cognitive processes,^8,42,43^ neural systems,^44–46^ and genetic factors.^47–51^ At each of these levels, some factors may be considered domain-specific and associated more strongly, or even uniquely, with behavioral performance in one domain (e.g., phonological processing and word-level reading). Others may be regarded instead as domain-general and implicated in performance across multiple domains (e.g., processing speed).^8,42,52–54^ The higher than chance co-occurrence of dyslexia and ADHD may be explained by shared domain-general factors (e.g., processing speed)^8,42,43,54,55^. Around these modal profiles of dyslexia and ADHD, there is substantial heterogeneity.^56–58^ The likelihood one or both disorders will manifest depends on the specific constellation and degree of factors implicated. This correlated liabilities^39^ and multiple deficits^26^ framework has proven useful also for understanding how transactions between children’s environments and their cognitive, neural, and genetic profiles dynamically vary risk for other disorders and patterns of co-occurrence.^25,59–62^.

## Outcomes

Compared to individuals with SLDs affecting a single academic domain, those whose difficulties span multiple domains and/or have co-occurring difficulties with attention and anxiety experience greater challenges. As we acquire clarity about these co-occurring difficulties and their impact, we have opportunities for greater specificity in treatment and potential for more effective outcomes. This knowledge can also be prognostic about potential intervention effects.^63^ For instance, compared to those with SLD isolated to a single academic domain, those with SLDs affecting multiple domains demonstrate more extensive and pronounced profiles of cognitive difficulties,^64,65^ resulting in earlier,^66,67^ more severe, and more persistent difficulties in each affected domain.^5,17,64,68–71^ This can render interventions less effective for individuals with co-occurring difficulties.^23^ For instance, in a randomized-controlled trail of a mathematics intervention for U.S. third-grade students with SLD, effects strongly favored those with SLD isolated to mathematics than those with SLD affecting mathematics and reading (ESs: 0.31–1.29).^72^

Similarly, individuals with SLD in reading and co-occurring ADHD exhibit on average more ADHD symptoms^54^ and greater academic,^54,55,73–75^ social,^74^ and behavioral difficulties.^74,76^ Moreover, these relative difficulties remain stable over the elementary grades.^55,74,75^ Studies have also shown that early attention difficulties predict later reading difficulties,^77,78^ and that behavioral attention negatively moderates immediate and long-term reading intervention effects.^79^

Compared to typically developing readers, those with reading difficulties have a higher risk for anxiety disorders,^80,81^ even after controlling for inattention.^82–84^ Although much has been speculated, little is known about why difficulties with reading and anxiety co-occur^80^ or how performance in one domain affects performance in the other.^85,86^ Several longitudinal investigations with elementary-level children have found a negative, bidirectional relation between reading and anxiety.^85,87,88^ However, results are mixed^89,90^ and the relation has been shown to vary across dimensions of reading (e.g., word-level reading, reading comprehension) and anxiety (e.g., separation, harm avoidance).^85,87,91,92^ Consistently stronger effects are found, though, between reading and reading anxiety than generalized anxiety.^87,88,91^ Much further investigation is needed to understand how anxiety, generalized or reading-specific, interacts with reading intervention outcomes, and whether reading interventions can reduce different forms of anxiety.

## Interventions for addressing co-occurring disorders

Effectively addressing the needs of individuals with SLD who have co-occurring disorders requires a comprehensive approach to intervention planning and implementation. We will focus here primarily on school-based academic and behavioral interventions. Many of these treatments are appropriate for clinical settings as well. We briefly describe pharmacological interventions that require input from and coordination with professionals and stakeholders outside of schools.

**Fig. 1:**
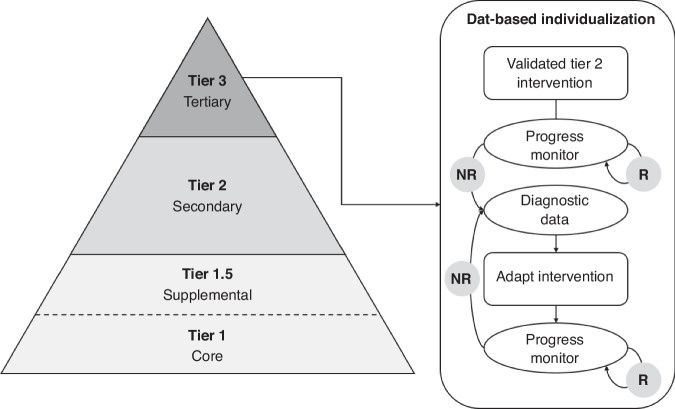
Multi-tiered systems of support framework. Data-Based Individualization process adapted from National Center on Intensive Intervention (2013). NR Not responsive to intervention, R Responsive to intervention.

Over the past 20 years, school-based academic, behavioral, and social-emotional interventions in the US have coalesced under the framework of multi-tiered systems of support (MTSS).^93^ As illustrated in Fig. 1, MTSS comprises three layers, or tiers, of preventive interventions: universal (Tier I), secondary (Tier 2), and tertiary (Tier 3).^94^ In practice, there is great variability in the number of components (i.e., academic, behavioral, social-emotional) and tiers that are implemented, as well as the specific interventions, measures, and procedures that are used.^95^ What we describe in the following sections should be regarded, then, as general principles rather than a standardized approach to MTSS implementation.

## Tier 1

Tier 1 is synonymous with general education.^96^ The intent is to prevent negative educational outcomes and to promote positive social-emotional development^97,98^ through interventions aimed at all students, including those with SLD and other disabilities. Indeed, the majority of US students with disabilities (65%) spend most ($\ge$80%) of their instructional days in general education,^3^ receiving the bulk of their instruction from general rather than special educators. It is imperative, then, that general education environments be designed to ensure maximal effectiveness and accessibility, with supports embedded for students with disabilities.^99^ To this end, Tier 1 practices should be aligned with validated principles of learning and instruction.

### Practice guidelines

Evidence-based instructional principles have been summarized in practice guides published by the US Department of Education’s Institute of Education Sciences (IES). Each IES practice guide provides recommendations within a particular subject area (e.g., foundational reading). For each recommendation, the level of supporting evidence is reported along with detailed suggestions for implementation. For instance, in the two practice guides for K-3 reading, nine recommendations are provided for teaching word-level reading and comprehension skills.^100^ Practice guides have also been developed for Tier 1 instruction in mathematics, writing, adolescent literacy, and behavioral interventions.

Although these guidelines do not constitute formalized interventions, they are intended to provide educators with clear, evidence-based parameters for instructional design and implementation. It is not expected that even when these guidelines are followed the needs of all students will be fully met. By following these guidelines, though, the extent and severity of difficulties may be mitigated. Moreover, these recommended practices are intended to provide solid foundations upon which more intensive and individualized support may be layered.

### Interventions

Much research has examined the efficacy of Tier 1 academic,^101–103^ behavioral,^104,105^ and social-emotional interventions.^106–109^ A comprehensive review of this work is beyond the scope of this article. We instead discuss consistent features. Detailed reviews of many Tier 1 interventions may be found, though, at the What Works Clearinghouse (https://ies.ed.gov/ncee/wwc/) and Evidence for ESSA (https://www.evidenceforessa.org/) websites.

Core Tier 1 interventions are intended to provide comprehensive approaches for academic, behavioral, or social-emotional support. These may be specific to a content area (e.g., reading) or part of comprehensive school-wide approaches that target performance in multiple domains (e.g., academic, social-emotional) or content areas (e.g., reading, math). Supplemental, or Tier 1.5,^110^ interventions layer upon core interventions, often targeting a more focused set of content and skills. Whereas some are designed to closely complement the content and structure of the core program, others are more unaligned.^111,112^ The content and structure of supplemental programs varies widely. However, several key features can be found in many, such as computer-assisted programs^101,113^ and cooperative learning.^114^

As reflected in the guides noted above, generalized practices have been induced from research on core and supplemental Tier 1 interventions. It is important to note, though, that research is unequally distributed across domains; that effects of specific programs vary considerably by domain, population, and outcome; and that initially promising effects often fade over time.^115–118^ Furthermore, most Tier 1 interventions target performance in a single domain. Although these may be combined within an integrated prevention system,^95,119,120^ such efforts can prove difficult to implement.^121,122^ Accordingly, this first layer of preventative support may not address the full range of a student’s potential difficulties.

## Tier 2

Tier 2 intervention is intended to reduce the prevalence and severity of educational difficulties by providing temporary support to selected groups of students. Students are selected for Tier 2 interventions based on screening data. A student’s responsiveness to Tier 2 intervention is used to determine whether more intensive support is needed (i.e., Tier 3). This information can also be used as part of a special education eligibility evaluation for SLD.^123–125^

Tier 2 academic, behavioral, and social-emotional interventions have been the focus of considerable research that extends beyond the scope of this article. We will instead focus on reading interventions. We will first discuss general findings from studies at the elementary and middle/high school levels. We will then turn to approaches that may be taken to support students with reading difficulties that co-occur with difficulties in mathematics, attention, and anxiety. Note that these studies include a mix of students with and without formal disability diagnoses.

### Reading interventions

The efficacy of elementary-level Tier 2 reading interventions has been the subject of much research. Gersten and colleagues^126^ summarized this work in an IES practice guide. Among their recommendations, they found strong evidence that Tier 2 reading interventions should entail intensive, systematic instruction on up to three foundational reading skills (e.g., phonemic awareness, letter sounds, listening comprehension). Moreover, they recommended that interventions should be delivered in small, homogenous groups $\ge 3$ timer per week for 20–40 min per session, and that skills should be built gradually, with ample opportunities for students to practice and receive feedback.

A meta-synthesis of 14 subsequent meta-analyses and systematic reviews of Tier 2 literacy interventions for K-5 students, also found consistent evidence that explicit and systematic interventions focused on word-level reading and reading comprehension are likely to improve student performance in word-level reading, and to a lesser extent reading comprehension.^127^ At the K-3 level, the largest review involved 72 studies.^128^ Interventions ranged from 15 to 99 sessions, largely involved K-1 students, and primarily (87.5%) focused on word-level reading. For both word-level reading and reading comprehension outcomes, average effects were larger for unstandardized than standardized measures. Effects were not moderated by intervention type, grade, implementer, or group size.

Several additional factors are important to note. First, effects are on average stronger for reading interventions that involve multiple rather than single components,^127^ likely reflecting the complexity of reading and the difficulties students experience. Second, dosage effects appear nonlinear. A meta-analysis of 26 K-3 reading interventions found that effects increased linearly until ~40 g of intervention, at which point they began to decrease.^129^ Similarly, in a meta-analysis of 16 studies examining the effects of phonemic awareness instruction, diminishing returns were found after 10 g of instruction.^130^ Finally, interventions delivered in the upper elementary grades (3-5) generally yield smaller effects.^127^ In a meta-analysis of 33 studies, an average ES of 0.22 standard deviations was found for foundational reading skills (i.e., phonemic awareness, phonics, fluency) and 0.21 for comprehension (vocabulary, comprehension).^131^ Effects were again larger for unstandardized (ES = 0.83) versus standardized (ES = 0.09) measures.

In contrast with intervention research at the early elementary level, less research has examined the efficacy of reading interventions for students in middle and high school. A summary of reading intervention in grades 4–9 is provided in an IES practice guide with strong evidence reported for developing students’ facility with decoding complex multisyllabic words and fluent reading of extended text passages.^132^ Strong evidence was found for building students’ word and world knowledge, providing frequent opportunities to ask and answer text-based questions, and apply strategies to determine the gist of short sections of text and to monitor their understanding. Moderate evidence supported the recommendation that students practice comprehending challenging texts identified as “stretch texts” containing complex ideas and information.

### Reading and attention

When students present with reading and attention difficulties, it is important to distinguish whether the reading difficulties arise from skill deficits or are secondary to ADHD symptoms.^133^ In the latter case, students’ reading difficulties may improve merely by addressing their ADHD symptoms. However, if they have fallen behind, reading remediation may still be required. When difficulties arise due to skill deficits, reading intervention is recommended.^134^ Reading intervention alone is unlikely to improve ADHD symptoms, though, and may be comprised if ADHD symptoms are not addressed. Accordingly, supporting students with co-occurring reading and attention difficulties will likely require a multimodal plan consisting of reading intervention and pharmacological and/or non-pharmacological interventions for ADHD.^135^

#### Reading intervention

Tier 2 reading interventions have been shown to be effective for children and adolescents with co-occurring reading and attention difficulties.^136,137^ A meta-analysis of 18 group-design studies found that interventions targeting word-level reading yielded greater benefits than those focused on reading comprehension.^137^ Furthermore, training reading skills beyond word-level reading did not produce incremental benefits over word-level reading alone. Nor were there benefits from adding pharmacological or behavioral interventions. Effects were not moderated by age, reading outcome, or alignment between intervention and outcome measure, but were smaller for studies that used more rigorous methods for diagnosing ADHD, involved students in self-contained settings, and that had higher dosages. In sum, large effects (ES = 1.11) may be expected on standardized reading outcomes from word-level reading interventions delivered for $\ge$30 g in regular classrooms to students with rigorously diagnosed ADHD and reading difficulties. Evidence is weaker for the efficacy of interventions targeting reading comprehension. A systematic review of 14 single-case design studies similarly found that word-level reading instruction is effective at improving reading outcomes.^136^

#### Non-pharmacological and pharmacological interventions

A wide range of non-pharmacological interventions (e.g., behavioral parent training, social skills training, cognitive training, neurofeedback, diet modification) have been developed to reduce ADHD symptoms and to improve academic, social, and behavioral performance for students with ADHD.^134,138^ Systematic reviews and meta-analyses of these interventions in school-based settings have shown that some can yield moderate to large improvements on academic and behavioral performance.^139^ Evidence is strongest for behavioral therapy that involves training adults and behavioral interventions that target skill development through repeated practice and feedback.^140,141^ For educational outcomes, the most effective interventions are those that are implemented in schools.^142^ However, most intervention studies have involved small samples of students with highly educated parents,^141^ focused primarily on the elementary rather than secondary grades,^143^ and have not examined effects in reading specifically, particularly for students with co-occurring reading difficulties.^139^

Stimulant and non-stimulant medications are approved by the U.S. Food and Drug Administration for treating ADHD in children aged 6 and older. There is strong evidence that they are safe and efficacious for reducing pediatric ADHD symptoms.^140,144^ Evidence is mixed, though, for their efficacy at improving reading outcomes for children and adolescents with ADHD and co-occurring reading difficulties. In a systematic review of 14 pharmacological intervention studies, Froehlich and colleagues^145^ examined the effects of methylphenidate (MPH) (stimulant) and atomoxetine (non-stimulant) on ADHD symptoms, neuropsychology, and reading performance for individuals with ADHD with and without a co-occurring reading SLD (ADHD-RD). MPH and atomoxetine effectively reduced parent- and teacher-reported ADHD symptoms both for children with ADHD and ADHD-RD. For both groups, MPH, but not atomoxetine, produced improved performance on a variety of neuropsychological measures (e.g., sustained attention, rapid automatize naming). Similarly, whereas MPH produced mixed effects on measures of reading for children ADHD-RD, atomoxetine provided little benefit.

#### Summary

There is strong evidence that WLR interventions are effective for improving WLR outcomes, but not RC outcomes, for students with ADHD and co-occurring reading difficulties. There is also strong evidence that pharmacological interventions can reduce ADHD symptoms for this population. There is also evidence that pharmacological interventions can improve neuropsychological performance for children with ADHD and co-occurring reading difficulties. Such improvements could potentially yield improved academic performance. However, the current evidence for this is weak.^146,147^ Evidence is also weak for the efficacy of reading comprehension interventions or pharmacological interventions at improving reading outcomes. Moreover, there is presently no evidence that reading interventions effectively improve ADHD symptoms. Therefore, consistent with clinical practice guidelines, multimodal interventions are likely necessary to address the complex needs of individuals with co-occurring ADHD and reading difficulties.

### Reading and mathematics

We are aware of only two studies that have examined the effects of reading interventions for students with co-occurring reading and mathematics difficulties. We have provided additional information about these and other studies we have reviewed in this article in Table S1. In the first, US first-grade students (*n* = 277) with isolated difficulties in word-level reading ($\le$25th percentile on word-level reading) or combined difficulties in word-level reading and mathematics calculations ($\le$25th percentile on word-level reading and calculations) were randomly assigned, in approximately even numbers, to a business-as-usual (BAU) control condition or to one of two tutoring interventions: (1) reading only or (2) reading and mathematics.^5^ Both interventions were delivered by researchers for 20–24 weeks, 3× per week, and 30–45 min per session. In both interventions, reading instruction comprised seven activities per session on sight-word recognition (e.g., *the*), decoding, spelling, and fluency. The mathematics interventions focused on strategic counting and number combination strategies during a brief, timed flash card activity.

On post-intervention calculations measures, students in the reading and mathematics condition outperformed those in the BAU (Effect Size [ES] = 0.60) and reading only conditions (ES = 0.57). Interestingly, though, whereas students in the reading only condition did not outperform those in the BAU condition on post-intervention word-level reading measures, those in the reading and mathematics condition did (ESs = 0.39 to 0.56). Calculations fluency fully mediated intervention group differences on word-level reading outcomes and partially mediated group differences on calculations outcomes.

In the second-grade study, students (*n* = 221) with co-occurring reading comprehension and word-problem solving difficulties were randomly assigned to BAU or interventions in reading or mathematics.^148^ The interventions were designed to support cross-domain transfer by leveraging parallels between reading comprehension and word-problem solving. For instance, both interventions included strategies for comprehending compare-contrast and cause-effect texts through close reading, analytical reasoning, text-structure vocabulary, and diagraming. Furthermore, one of the five units focused explicitly on cross-domain transfer.

On domain-aligned post-intervention measures (e.g., reading intervention → reading outcome), students in both interventions outperformed those in the BAU condition (Reading ESs: 0.84–1.16; Math ESs: 1.05–1.36). Furthermore, students in both interventions evidenced cross-domain transfer of learning with smaller but still large effects for reading → math (ESs: 0.41–0.68) and math → reading (ESs: 0.54–0.91). Students in both interventions performed comparably on post-intervention reading measures. However, those in the mathematics condition significantly outperformed those in the reading condition on mathematics outcomes.

#### Summary

Results from these two studies provide promising initial evidence for understanding how to effectively support young children with co-occurring reading and mathematics difficulties. Intervening upon factors that underlie the covariance between reading and mathematics performance appears particularly beneficial, and perhaps necessary, for affecting improvements in both domains. Future research is needed to understand how these results hold under replication and extension to older and populations and different subdomains of reading and mathematics.

### Reading and anxiety

For individuals with co-occurring reading and anxiety disorders, intervention should target both domains. This may be approached through separate reading interventions and anxiety management programs or in combination. Below we discuss findings from both approaches.

#### Anxiety management

Clinical practice guidelines recommend combinations of cognitive-behavioral therapy and medications for treating different manifestations of childhood and adolescent anxiety disorders.^149^ A recent scoping review found 21 studies that had examined effects of non-pharmacological anxiety management interventions on anxiety and academic outcomes specifically for students with SLDs.^150^ There was considerable variation in the interventions’ focus, goals, theoretical framework, and methods. Some of these focused on teaching anxiety management skills (e.g., mindfulness, relaxation), others on academic remediation (e.g., mathematics, reading), and others on combinations of these two approaches. Although 73% of the studies reported reductions in anxiety and improved academic performance, effects were mixed and few (38%) used randomized designs. Furthermore, only several specifically examined reading outcomes, for which findings were mixed.

The effects of anxiety management interventions on reading and anxiety outcomes have been the subject of several case studies and one group design study.^151–153^ In the latter, Canadian students (*n* = 36), aged 9–12 years, were assigned to BAU or anxiety management conditions.^152,153^ The intervention comprised a four-stage muscle relaxation procedure delivered over 12 30-min sessions twice a week over 6 weeks. Students in the intervention condition did not significantly outperform those in BAU on post-intervention measures of word-level reading or reading comprehension or show improvements in anxiety during reading or non-reading tasks.

#### Reading and anxiety management

Several studies have examined the effects of combined reading and anxiety management interventions. In a US study, third- and fourth-grade students (*n* = 128) with reading difficulties were randomly assigned to one of three groups: control group, a reading and anxiety management group (RANX), or a reading and mathematics group (RMATH).^154^ Over the 2 years, both interventions included 150 30-min lessons delivered 4–5 times per week in groups of 2–5 students. In both interventions, reading instruction targeted word-level reading, fluency, vocabulary, and comprehension. The anxiety management component was integrated within the reading intervention time and comprised 10 evidence-based cognitive-behavioral practices aimed at managing anxiety during reading. Skills focused on recognizing different feelings, practicing relaxation and stress management, and recognizing and managing anxious and unhelpful thoughts. During Year 1, ~5 min of anxiety management training was provided each lesson. In Year 2, this was reduced to about every third lesson.

Accounting for differences at the end of Year 1, RANX students significantly outperformed those in BAU (ES = 1.22) and RMATH (ES = 0.77) on one reading comprehension measure at the end of Year 2. There were no significant differences among the groups, though, on other measures of reading comprehension or word-level reading. Reading anxiety moderated the effect of RANX on the word-level reading measure, such that effects were stronger for students with lower reading anxiety.^154^ Reading anxiety outcomes were mixed, with insignificant effects after Year 1, but improved general and social anxiety for RANX students after 2 years of intervention.^155^

#### Summary

Although there is strong evidence that pharmacological and non-pharmacological anxiety management interventions can effectively reduce different forms of childhood and adolescent anxiety, there is currently no evidence that anxiety management interventions alone are effective at improving reading performance. Combined reading and anxiety management interventions show promise for improving outcomes in both domains. However, findings from this nascent literature are mixed. Additional intervention studies are necessary before any firm conclusions can be drawn. As this work proceeds, precision is needed to understand relations between various dimensions of reading and anxiety and how to optimally time, sequence, and coordinate behavioral and pharmacological interventions to address specific combinations of reading and anxiety difficulties.

## Tier 3

Tier 3 interventions are characterized by intensive, individualized, and ongoing support for students with persistent learning, behavioral, and emotional difficulties.^156^ In some MTSS frameworks, this is synonymous with special education.^96^ However, as we have noted, support for students with disabilities should be provided across tiers.^99^ Different approaches have been proposed for providing intensive and individualized support.^157–159^ Common among these is the use of student progress monitoring data to inform decisions concerning the selection and adaptation of interventions. Here we will highlight data-based individualization (DBI), a framework that has been shown to be effective for individualizing reading,^160–162^ mathematics,^160,163^ and writing instruction.^164^

### Data-based individualization

DBI is problem-solving approach for intensifying academic and behavioral support.^165^ As illustrated in Fig. 1, DBI begins by selecting a validated intervention and one or more long-term goals (e.g., 1 year) based on the student’s academic and/or behavioral needs. Once the intervention commences, the student’s performance is regularly monitored (e.g., weekly) to evaluate progress toward their goal(s). If the student shows adequate progress toward their goal(s), the intervention proceeds unaltered. Alternatively, if the student does not progress adequately, diagnostic data are collected and used to adapt features of the intervention (e.g., dosage).^166^ Progress then continues to be monitored frequently. If the intervention again proves inadequate, additional diagnostic data are collected and further adaptations are made until the student reaches or exceeds their goal.^158^ The flexible design of this DBI should easily accommodate support for attention, anxiety, and other potential sources of learning difficulties. By frequently monitoring progress, the educator or clinician can test the efficacy of such instructional adaptations for their students. However, research is still needed to test the efficacy, feasibility, and acceptability of DBI for students with co-occurring difficulties and when implemented across multiple domains.

## Conclusions and future directions

School-based intervention research has primarily focused on difficulties within single domains. It is clear, though, that when a student experiences difficulties in one domain, they are likely to experience difficulties in another. The co-occurrence of learning and behavioral difficulties is an implicit driver of recent efforts to unify formerly disparate prevention systems focused on academic, behavioral, and social-emotional development. Coalescing these systems undoubtedly presents a complex and difficult undertaking. However, a unified system could have large implications for the prevention of difficulties not only within but between performance domains.

We discussed recent efforts to unify Tier 2 interventions for students with difficulties in reading that co-occurr with difficulties in mathematics, attention, and anxiety. Research in these areas is limited and uneven, greatly restricting the recommendations that can be drawn. Studies on interventions for students with co-occurring reading and attention difficulties has advanced the furthest, providing strong evidence supporting the efficacy of word-level reading interventions.^137^ Far less research has examined how to support students with difficulties in reading that co-occur with difficulties in mathematics or anxiety. Although there are promising findings in both areas, far more work is needed to replicate and extend these initial findings.

Beyond the three pairs of disorders we discussed, students may present myriad other patterns of educational strengths and difficulties. The subtyping framework we adopted is one approach for categorizing these patterns. There are of course alternatives.^167^ For instance, in the transdiagnostic approach, traditional categories (e.g., dyslexia) are replaced with performance dimensions (e.g., word-level reading) and functional descriptors (e.g., low word-level reading).^168,169^ This is intended to provide a more reliable classification system that better acknowledges the idiosyncratic ways difficulties manifest within and between performance domains. Future research should compare the relative benefits of this, and others approaches, for identifying and supporting students with learning difficulties.

Finally, nearly all the research we discussed involved elementary school students. Early prevention certainly holds the greatest potential for reducing the prevalence and severity of learning difficulties. Even when early interventions are provided, though, many students continue to require intensive support throughout their educational careers. It should also not be assumed that interventions found to be effective for children will be equally effective, or appropriate, or feasible for adolescents and adults. Research is greatly needed, then, to understand how to effectively support older students. More studies are also needed to understand the long-term effects of interventions,^115^ the optimal dosage and sequence supports within and between domains,^170^ and the effects of intervention alignment within and between prevention tiers.^171^

## Supplementary information

Supplementary Table S1
