# Supplementary material for: Interventions for children and adolescents with specific learning disability and co-occurring disorders
Source: Pediatr Res. 2025 Jul 18;98(7):2536–44. doi: 10.1038/s41390-025-04261-0 (PMC12875878; doi:10.1038/s41390-025-04261-0)
Supplement: Supplementary file 1 — Supplementary Table S1 [file 41390_2025_4261_MOESM1_ESM.pdf]

Table S1

Intervention Components

Study	Conditions	Number of Sessions	Sessions Per Week	Duration (in weeks)	Session Length (in mins.)	Group Size
			Reading Instruction and Mathematics Instruction			
Fuchs et al. (2013)	(a) BAU control, (b) reading intervention along, (c) reading intervention + number combination intervention	Not specified	3	20-24	30-45	NR
Fuchs et al. (2024)	(a) BAU control, (b) mathematics WPS intervention, (c) RC intervention	45	3	15	30	1
			Reading Instruction and Anxiety Management			
(Vaughn et al., 2022)	(a) BAU control, (b) reading intervention with anxiety management, (c) reading intervention with math fact practice.	150	4-5	75 (per year)	30	2-5

Note. BAU = business-as-usual, WPS = word-problem solving, RC = reading comprehension.

References

Fuchs, L. S., Fuchs, D., & Compton, D. L. (2013). Intervention effects for students with comorbid forms of learning disability: Understanding the needs of nonresponders. *Journal of Learning Disabilities*, *46*(6), 534–548. https://doi.org/10.1177/0022219412468889

Fuchs, L. S., Seethaler, P. M., Sterba, S. K., Fuchs, D., Cutting, L. E., Mancilla-Martinez, J., Martin, B., & Espinas, D. R. (2024). Transfer between reading comprehension and word-problem solving in children with comorbid difficulty via text-structure intervention. *Journal of Educational Psychology*, *116*(7), 1093–1111. https://doi.org/10.1037/edu0000911

Vaughn, S., Grills, A. E., Capin, P., Roberts, G., Fall, A.-M., & Daniel, J. (2022). Examining the effects of integrating anxiety management instruction within a reading intervention for upper elementary students with reading difficulties. *Journal of Learning Disabilities*, *55*(5), 408–426. https://doi.org/10.1177/00222194211053225
